# Will Hydroxychloroquine Still Be a Game-Changer for COVID-19 by Combining Azithromycin?

**DOI:** 10.3389/fimmu.2020.01969

**Published:** 2020-08-07

**Authors:** Chunfeng Li, Genhong Cheng

**Affiliations:** ^1^Institute for Immunity, Transplantation, and Infection, School of Medicine, Stanford University, Stanford, CA, United States; ^2^Department of Microbiology, Immunology, and Molecular Genetics, University of California, Los Angeles, CA, United States

**Keywords:** COVID-19, SARS- CoV-2, hydroxychloroquine, azithromycin (AZM), clinical trial

## Abstract

Recent small-scale clinical trials have shown promising results in the use of hydroxychloroquine, an FDA approved anti-malaria drug, for the treatment of COVID-19. However, large scale, randomized and double-blind clinical trials are needed to confirm the safety and efficacy of hydroxychloroquine in COVID-19 patients. Here, we review the progress of using hydroxychloroquine or chloroquine as anti-viral agents, failed clinical trials of chloroquine in treatment of dengue virus and influenza infection, and especially the mechanism of azithromycin in inhibiting viral replication, so as to shed light on the ongoing clinical trials and further researches of hydroxychloroquine on SARS-CoV-2 infected patients.

As of 6/3/2020, more than 6.3 million people across the world have been infected with SARS-CoV-2, with the death toll reaching 383,000 since the beginning of the outbreak in late December 2019 (https://coronavirus.jhu.edu/map.html). This pandemic has changed our daily life dramatically, especially of those who are directly infected. SARS-CoV-2 is a highly virulent newly emerging coronavirus belonging to the Betacoronavirus genus within the family of Coronaviridae, which includes four genera: Alphacoronavirus, Betacoronavirus, Gammacoronavirus, and Deltacoronavirus (1). SARS-CoV-2 and other four human coronaviruses (OC43, SARS-CoV, MERS-CoV, and HKU1) belong to the Betacoronavirus genus, while two other human coronaviruses NL63 and 229E, PDEV (porcine epidemic diarrhea virus) in pigs, TGEV (transmissible gastroenteritis virus) in swine, FCoV (feline coronaviruses), CCoV (canine coronaviruses), FRECV (ferret enteric coronavirus) belong to the Alphacoronavirus genus (2). Like SARS and MERS, SARS-CoV-2 was originally found in a bat reservoir, but was transmitted through a different intermediate host like pangolin (3).

## Hydroxychloroquine and Chloroquine Inhibit Virus Infection *in vitro* and *in vivo*

Currently there are no FDA approved drugs for COVID-19. Recently, Wang et al. reported that chloroquine can inhibit SARS-CoV-2 replication *in vitro* (4). Chloroquine is an FDA-approved anti-malarial drug. Hydroxychloroquine is a derivative of chloroquine. They have similar safety profiles and therapeutic effects. Hydroxychloroquine has also been widely used for the treatment of systemic lupus erythematosus (SLE) due to its anti-inflammatory function. Yao et al. found that hydroxychloroquine (EC_50_ = 0.72 μM) is more potent than chloroquine (EC_50_ = 5.47 μM) in inhibiting SARS-CoV-2 *in vitro* (5). It is well-established that chloroquine or hydroxychloroquine can inhibit different viruses *in vitro*, including encephalomyocarditis virus, Sindbis virus, influenza A virus, Newcastle disease virus, herpes simplex virus, vaccinia virus, VSV (6–8), also including, Chikungunya virus (CHIKV) (9), dengue virus (DENV) (10), Ebola virus (11), and the SARS-CoV (12). Our and other's data have also shown that chloroquine can suppress Zika virus (ZIKV) replication in different cells *in vitro* (13–16).

We have also used different mouse models like Balb/c mice, Ifnar1^−/−^ mice, neonatal mice, and pregnant mice to confirm the *in vivo* activity of chloroquine in inhibiting ZIKV (13). With the pregnant mouse model, we found that orally administrated chloroquine can prevent ZIKV infection in the fetal brain and rescue microcephaly caused by ZIKV infection. Our data also demonstrated that chloroquine can inhibit ZIKV replication more efficiently when delivered soon after infection (13). In agreement with our findings, Tan et al. found that chloroquine could inhibit enterovirus EV-A71 in mice more efficiently if administered before infection (17). Chloroquine can also inhibit other viruses *in vivo*, including human coronavirus OC43 (18), and influenza A H5N1 (19). However, chloroquine is ineffective in inhibiting Ebola virus (11, 20), Nipah virus (21), and influenza virus (22) *in vivo* due to multiple reasons, such as the ways and timing to administrate the drugs, the host animal model used, the replication kinetics of different viruses and so on.

## Clinical Trials of Hydroxychloroquine or Chloroquine in Patients With Other Infectious Diseases, and its Implications for COVID-19

In a clinical trial of DENV infected patients receiving chloroquine for 3 days beginning 72 h after infection (Table 1), chloroquine did not reduce viremia (23, 24). In another clinical trial of chikungunya virus infection, patients were given 600 mg chloroquine for the first 3 days and 300 mg for another 2 days (Table 1). The results showed that 11 out of 27 patients from both the test and control groups were negative for viral RNA test on day 3 and all patients were negative on day 6. Chloroquine also did not improve patient outcomes, but was associated increased risk of arthralgia at day 200 after treatment (9, 32). Finally, in a randomized, double-blind, and placebo controlled trial on the effect of chloroquine on prevention of influenza, healthy adults received chloroquine phosphate (500 mg/day for 1 week, then once a week for 11 weeks) or placebo were monitored for flu symptoms for 1 year (Table 1). Ultimately, 29 out of 738 (4%) placebo and 38 out of 724 (5%) chloroquine-treated subjects were infected with influenza (25). In these clinical trials, mild adverse events were only seen in chloroquine-treated groups.

**Table 1:** Clinical trials of hydroxychloroquine or chloroquine w/o azithromycin in patients with infectious disease.

**Drug**	**Target**	**Treatment**	**Duration**	**Trial design**	**Patient outcome**	**References**
Chloroquine	DENV	The regimen for CQ was 600 mg base (4 × 150 mg tablets) on enrolment to the study, then 600 mg on day 2 and 300 mg on day 3	3 days	Randomized, double blind, placebo controlled; CQ (*n* = 153) or placebo (*n* = 154)	Time to resolution of DENV viraemia and time to resolution of DENV NS1 antigenaemia; fever clearance time; dengue hemorrhagic fever	(23)
Chloroquine	DENV	500 mg chloroquine (300 mg base) BID	3 days	Randomized, double blind, placebo controlled; CQ (*n* = 9) or placebo (*n* = 18)	Duration of the disease or the degree and days of fever	(24)
Chloroquine	Chikungunya virus	600 mg chloroquine for the first 3 days and 300 mg for another 2 days	5 days	Randomized, double blind, placebo controlled; CQ (*n* = 27) or placebo (*n* = 27)	Duration of febrile arthralgia, decrease of viremia; arthralgia at day 200	(9)
Chloroquine	Influenza virus	500 mg/day for 1 week, then once a week for 11 weeks	12 weeks	Randomized, double blind, placebo controlled; CQ (*n* = 724) or placebo (*n* = 738)	Lab-confirmed influenza infection defined by specific symptoms and RT-PCR on nasal swabs, or a 4-fold increase in HA-inhibition titres over 12 weeks	(25)
Chloroquine w/o azithromycin	P. falciparum	600 mg base (4 × 150-mg base tablet) of chloroquine for 2 days and 300 mg base (2 × 150-mg base tablet) of chloroquine on day 3; w/o 1 g (2 × 500-mg tablet) of azithromycin;	3 days	Open label non-randomized; *n* = 64 for combination treatment; chloroquine only (*n* = 16); AZM only (*n* = 16)	Parasite counts	(26)
Chloroquine and azithromycin	P. falciparum	AZM 1,000 mg and CQ 600-mg base once daily for 3 days	3 days	Multi-Country Randomized; CQ and AZM combination (*N* = 227); mefloquine hydrochloride (*N* = 231)	P. falciparum parasite clearance rate	(27)
Hydroxychloroquine	SARS-CoV-2	400 mg/d (200 mg/bid) between day 1 and 5	5 days	Randomized, HCQ (*n* = 31) or placebo (*n* = 31)	Time to clinical recovery, clinical characteristics, and radiological results were assessed at baseline and 5 days after treatment	(28)
Hydroxychloroquine w/o azithromycin	SARS-CoV-2	HCQ: 200 mg, three times per day; AZM: 500 mg on day 1 followed by 250 mg per day for another 4 days	10 days for HCQ; 5 days for AZM	Open label non-randomized; HCQ (*n* = 20) or placebo (*n* = 16); 6 patients from HCQ group also received azithromycin	Viral load	(29)
Hydroxychloroquine and azithromycin	SARS-CoV-2	HCQ: 200 mg, three times per day; AZM: 500 mg on day 1 followed by 250 mg per day for another 4 days	10 days for HCQ; 5 days for AZM	Open label non-randomized; All patients received combination treatment	Viral load	(30)
Chloroquine	SARS-CoV-2	High dose: 600 mg CQ twice daily; Low dose: 450 mg twice daily on day 1 and once daily for 4 days	High dose for 10 days; Low dose for 5 days	Randomized; High dose (*n* = 41), low dose (*n* = 40)	Reduction in lethality	(31)

For antiviral treatment of acute infectious disease, the therapeutic time window is a critical factor controlling patient outcomes. Based on findings from China, the median period of viral shedding is 20.0 days and could be detected up to 37 days in COVID-19 survivors (33). A study from Singapore showed that shedding of SARS-CoV-2 from nasopharyngeal aspirates of patients was detected up to 24 days after symptom onset (34). The median period from onset of symptoms to ARDS (Acute Respiratory Distress Syndrome) is around 8–11 days for COVID-19 patients and the duration of fever is around 8–12 days in survivors (33), which is longer than 2–7 days for influenza, Zika, or dengue fever (35). This characteristic of COVID-19 provides a longer and better therapeutic window for antiviral drug treatment before reaching the “point of no return.” To reach this goal, early diagnosis and fast testing, which means a turn-around time of <24 h, are “bottlenecks” for most of hospitals in the midst of the crisis, especially for developing countries that do not have enough detection equipment, reagents, and professionals.

## Clinical Trials of Hydroxychloroquine or Chloroquine in COVID-19 Patients

Recently, Yao et al. found that, based on the difference of IC_50_ of hydroxychloroquine and chloroquine to SARS-CoV-2, the predicted physiologically-based pharmacokinetics (PBPK) of hydroxychloroquine in the human lung are more advantageous than those of chloroquine in inhibiting SARS-CoV-2 *in vivo*. As a result, hydroxychloroquine was suggested in clinical trials to combat COVID-19 by the authors (5). In a preprint manuscript, Chen et al. tested the efficacy of hydroxychloroquine [400 mg/d (200 mg/bid) between day 1 and 5] with 31 patients, and another randomized 31 patients served as controls (Table 1). The patients were enrolled after positive RT-PCR test and CT scan with pneumonia. Control patients received standard treatment only. Body temperature recovery time (2.2 vs. 3.2 days) and cough remission time (2.0 vs. 3.1 days) were significantly reduced in hydroxychloroquine treated patients (28). Gautret et al. also evaluated the efficacy of hydroxychloroquine on COVID-19 patients (Table 1). In their trial, 20 patients in Marseille were orally administrated hydroxychloroquine sulfate 200 mg, three times per day for 10 days soon after testing positive for SARS-CoV-2, while 16 patients from Marseille or other nearby cities who refused the treatment served as controls. Six patients out of the 20 hydroxychloroquine-treated patients also received azithromycin (AZM) orally (500 mg on day 1 followed by 250 mg per day for another 4 days) to prevent bacterial infection. Six days later, all patients who received hydroxychloroquine and azithromycin tested negative for SARS-CoV-2 by RT-PCR (virologic cure), while 57.1% of patients treated with hydroxychloroquine, and 12.5% in the control group were negative (29). They also showed in another clinical study of 80 COVID-19 patients that hydroxychloroquine combined with AZM could reduce nasopharyngeal viral load significantly (83% negative at Day 7, around 12 days post-onset of symptoms, and 93% at Day 8 tested by RT-PCR) (Table 1) (30), compared to median 20 days of viral shedding in other COVID-19 patients (33, 34). In contrast, there are other small clinical trials showing hydroxychloroquine alone or combined with azithromycin could not improve viral suppression or outcomes in COVID-19 patients (36, 37). However, these cohorts are very small, and the testing methods, patient status, and supporting care from different hospitals are different. Therefore, large-scale, randomized, double-blind, placebo controlled trials are essential to determine the efficacy and safety of hydroxychloroquine and its combination with azithromycin in controlling COVID-19. These trials are very important for patients in countries such as the United States that are currently in crisis, and countries such as India, and South Africa, which may suffer COVID-19 outbreaks in the near future. Gathering more robust data now about COVID-19 will make us more prepared in the future.

## Azithromycin may Contribute to Hydroxychloroquine Function Through Different Mechanisms

It remains to be seen whether hydroxychloroquine combined with AZM is more effective in treating COVID-19 efficient than hydroxychloroquine alone, and the *in vivo* mechanisms by which this potential synergy occurs is unknown. If yes, what's the potential mechanism *in vivo*? AZM is a macrolide antibiotic that can broadly inhibit gram-positive and negative bacteria targeting protein synthesis of bacterial ribosomes (38). Mortality and morbidity caused by bacteria pneumonia with coinfections maybe reduced by AZM in patients infected by Spanish flu (39) and SARS-CoV-2 (33). We and other labs found that AZM can suppress Zika virus *in vitro* (40, 41). AZM enhances the type I and type III interferon signaling pathway and its downstream interferon stimulated genes (ISGs) response triggered by Zika virus infection *in vitro*. Furthermore, AZM increases expression of phosphorylated TBK1 and IRF3 to prime the host cell to respond to viral infection. AZM upregulates the interferon response induced not only by ZIKV, DENV, and polyI:C (RNA), but also by polydA:dT (DNA). These data show that AZM can be used as a broad antiviral reagent (40). AZM has also been found to inhibit rhinoviruses by enhancing Type I interferon (IFN-I) responses in bronchial epithelial cells (42, 43). Additionally, AZM has been shown to inhibit Ebola virus *in vitro*, however the mechanism is not understood (44). In another study, Dr. Raoult's lab showed that AZM has synergistic effects with hydroxychloroquine to inhibit Sars-CoV-2 *in vitro*, although the anti-SARS-CoV-2 activity of AZM alone is not very promising in this system (45). Moreover, AZM was used to treat infections with an inflammatory component due to its anti-inflammatory properties (46). With regard to pneumonia and cytokine storm in severe COVID-19 patients, the anti-inflammatory function of azithromycin, and hydroxychloroquine combined therapy can achieve better patient outcomes. AZM was found to kill senescent human fibroblasts, which may express more ACE2 and CD26 (47). ACE-2 (angiotensin-converting enzyme 2) is receptor for SARS-CoV-2, and CD26 [also known as dipeptidyl-peptidase IV (DPP4)] may interact with S protein of SARS-CoV-2 (1, 48). Hydroxychloroquine can reduce expression of lysosomal enzyme beta-galactosidase (Beta-Gal), a widely-recognized marker of senescence. Hydroxychloroquine can also reduce salivary and serum levels of IL-6, a key component of the senescence-associated secretory phenotype (SASP) in chronic inflammatory diseases, such as Sjögren's syndrome (49). High levels of IL-6 in plasma is also related to severe pathogenesis in COVID-19 patients (50, 51). AZM may block SARS-CoV-2 infection through different mechanisms and have synergistic effects with hydroxychloroquine *in vitro* and *in vivo*, which needs to be investigated in future.

AZM and hydroxychloroquine have additional advantages: they are affordable for low-income populations, can be orally administered, have very good safety profiles, and very little drug-drug interactions (52). AZM belongs to FDA pregnancy category B drug, meaning that animal studies showed no adverse effects on pregnancy (53, 54). Hydroxychloroquine and chloroquine are pregnancy category C drugs, meaning that there is a potential risk for the fetus based on animal studies, but if the benefits outweigh the risks, they may continue to be used during pregnancy to treat related symptoms. An acceptable safety profile was achieved by combining azithromycin 1,000 mg and chloroquine 620 mg/day for 3 days in 1,446 pregnant women in sub-Saharan Africa (55). Azithromycin can suppress *P. falciparum* (*Plasmodium falciparum*) *in vitro* and *in vivo* (Table 1). In randomized clinical trials, AZM can enhance the efficacy of chloroquine for the treatment of acute uncomplicated malaria caused by *P. falciparum* in India and Africa (26, 27). Pharmacokinetic studies showed that after oral administration of AZM 1,000 mg/day for 3 days, the concentration of AZM in lung reaches peak concentrations of 17.85 μg/g (around 24 μM) (56). The half-lives of AZM in the lung and bronchial wash are 133.32 and 70.5 h, respectively. With that concentration, AZM may inhibit SARS-CoV-2 *in vitro* and *in vivo* through the mechanisms mentioned above, which needs to be tested thoroughly in the future.

During revision of this review, prospective observational studies of safety, and efficacy of hydroxychloroquine with or w/o AZM in COVID-19 patients were conducted by different groups with different cohorts. Huang et al. showed that chloroquine is beneficial for COVID-19 patients in China (57), while Geleris et al. found that hydroxychloroquine w/o AZM was not related to either a greatly lowered or an increased risk of the composite end point of intubation or death (58, 59). The major differences between these two studies are that patients from the former study are much younger (average age of the patients ~45 vs. ~57), and fewer with severe symptoms that requires ventilation compare to those from the later study (57). While other retrospective studies found that hydroxychloroquine with or w/o AZM may be related to more mortality in COVID-19 patients (59, 60), which may due to the fact that hydroxychloroquine was more likely to be prescribed to patients with more severe disease under emergency use authorization. These data are also indicating that hydroxychloroquine with or w/o AZM may be beneficial for mild or moderate but not severe patients. However, we note that there are potential risks associated with hydroxychloroquine/chloroquine for cardiovascular disorders and ophthalmological damage if used at high dose, for example 600 mg chloroquine twice daily for 10 days, which is 4–5 times greater than the American Academy of Ophthalmology (AAO) recommendation (31, 61, 62). As azithromycin and hydroxychloroquine can work as senolytics *in vitro*, we should be more cautious about cardiovascular disorder as cardiac cells become more senescent with aging (63, 64). More recently, Mercuro et al. found that hydroxychloroquine with or w/o AZM increased risk of corrected QT (QTc) prolongation and cardiac arrhythmias in COVID-19 patients (65). Combined hydroxychloroquine and AZM use should follow the physician's instructions, and cardiovascular and ophthalmological toxicity should be monitored during and after the short-term treatment, especially for older patients.

At the same time, the death toll in some countries, especially in older populations, remains very high. The efficacy of hydroxychloroquine in severe patients remains unknown. Many undergoing efforts are also focused on testing drugs like Remedesivir (ClinicalTrials.gov Identifier: NCT04292899) (66), HIV inhibitors (Favipiravir) (67), IL-6 antibodies [Actemra® (tocilizumab), NCT04320615], plasma from recovered patients (68), and so on. Additionally, screening of other FDA approved drugs or drugs under clinical trials may provide us more promising drug candidates for COVID-19 (69, 70).

In conclusion, SARS-CoV-2 is a different and challenging pathogen in many ways. It is more contagious than SARS-CoV and causes a higher fatality rate compared to the 1918 influenza virus. However, its long period of viral shedding and clinical symptoms like fever and cough actually provide a better therapeutic window for antiviral treatment. Small-scale clinical data has shown that hydroxychloroquine can improve patient outcomes and that concurrent azithromycin treatment may have synergistic effects. There are many different mechanisms by which AZM can contribute to the efficacy of hydroxychloroquine *in vitro* and *in vivo*, which need to be investigated carefully in the future. It is possible that different categories of patients, such as young vs. old, and mild vs. severe, may respond to hydroxychloroquine with or w/o AZM differently. Attention should be paid to the side effects of hydroxychloroquine w/o AZM in COVID-19 patients, especially in the older patients. We argue that large-scale, randomized, and double-blind clinical trials are still urgently and essentially needed to test the safety and efficacy of hydroxychloroquine w/o AZM in COVID-19 patients, and we should continue to find and test other potential treatments for COVID-19 and other emerging infectious diseases.

## Author Contributions

All authors listed have made a substantial, direct and intellectual contribution to the work, and approved it for publication.

## Conflict of Interest

The authors declare that the research was conducted in the absence of any commercial or financial relationships that could be construed as a potential conflict of interest.
